# Fast-Food Consumption and Obesity Among Michigan Adults

**Published:** 2011-06-15

**Authors:** Beth Anderson, Sarah Lyon-Callo, Christopher Fussman, Gwendoline Imes, Ann P. Rafferty

**Affiliations:** Michigan Department of Community Health; Michigan Department of Community Health, Lansing, Michigan; Michigan Department of Community Health, Lansing, Michigan; Michigan Department of Community Health, Lansing, Michigan; Ann P. Rafferty was affiliated with the Michigan Department of Community Health during data analysis and initial drafting

## Abstract

**Introduction:**

Consumption of meals eaten away from home, especially from fast-food restaurants, has increased in the United States since the 1970s. The main objective of this study was to examine the frequency and characteristics of fast-food consumption among adults in Michigan and obesity prevalence.

**Methods:**

We analyzed data from 12 questions about fast-food consumption that were included on the 2005 Michigan Behavioral Risk Factor Survey, a population-based telephone survey of Michigan adults, using univariate and bivariate analyses and multivariate logistic regression, and compared these data with data on Michigan obesity prevalence.

**Results:**

Approximately 80% of Michigan adults went to fast-food restaurants at least once per month and 28% went regularly (≥2 times/wk). Regular fast-food consumption was higher among younger adults (mostly men) but was not significantly associated with household income, education, race, or urbanicity (in a multivariate framework). The prevalence of obesity increased consistently with frequenting fast-food restaurants, from 24% of those going less than once a week to 33% of those going 3 or more times per week. The predominant reason for choosing fast food was convenience. Although hypothetically 68% of adults who go to fast-food restaurants would choose healthier fast-food items when available, only 16% said they ever use nutritional information when ordering.

**Conclusion:**

The prevalence of fast-food consumption is high in Michigan across education, income, and racial groups and is strongly associated with obesity. Making nutritional information at fast-food restaurants more readily available and easier to use may help consumers to order more healthful or lower-calorie items.

## Introduction

The prevalence of obesity in the United States has increased dramatically during the past few decades and is now a major public health concern (1). Michigan is no exception; the prevalence of adult obesity increased from 18% to 26% from 1995 through 2005 (2). Concurrent with this increase in obesity has been an increase in calorie intake. Results from Kant and Graubard's (3) analysis of data from the National Health and Nutrition Examination Survey (NHANES) indicate increases in quantity and energy density of foods consumed in the United States between 1976 to 1980 (NHANES II) and 1999 to 2002 (NHANES III), and adjusted estimates from the US food supply indicate that per capita calorie intake increased by more than 300 kilocalories (kcal) among the entire population from 1985 through 2002 (4). Many factors, including behavioral, environmental, cultural, and socioeconomic influences (1), affect people's food choices. These choices, which affect the balance of energy intake, combined with genetic and metabolic factors, determine body weight and composition (5).

Since the 1970s the consumption of food eaten away from home has also increased in the United States (6-8). Eating out may lead to overconsumption and increase the risk of obesity in part because of larger portion sizes, high–energy-dense foods, and increased variety and preferred taste of the foods (9-11). Fast-food consumption in particular has been associated with poor diet quality and adverse dietary factors related to obesity, including higher intakes of calories, fat, saturated fat, and sugar-sweetened drinks (12,13). Fast food is associated with higher body mass index (BMI), weight gain, and less successful weight-loss maintenance (12,14-16). In addition, increased exposure to fast-food restaurants at the neighborhood level is associated with poorer diet quality and increased obesity (17,18). Fast-food consumption is a trend that is still rising; quick-service restaurants are expected to post sales of $164.8 billion in 2010, a 3% increase from 2009 (19).

Few studies have estimated fast-food consumption at the population level. One set of studies that used data from the Continuing Survey of Food Intakes by Individuals has documented an increase in fast-food consumption nationally (ie, 1 in 6 US adults reported eating fast food during a 2-day nutritional profile during 1989-1991, whereas 1 in 4 adults reported fast-food consumption during 1994-1996) (12). Data from a population-based telephone survey in Minnesota estimated that 51% of adults ate fast food at least once a week (20), and in Canada, one-fourth of respondents to the national 2004 Canadian Community Health Survey aged 19 years or older reported eating fast food on the day before the interview (21).

The main objective of this study was to examine the population-based prevalence and characteristics of fast-food consumption among Michigan adults aged 18 to 64 years. A secondary objective was to investigate the association between regular fast-food consumption and obesity prevalence.

## Methods

We analyzed data from the 2005 Michigan Behavioral Risk Factor Survey (MiBRFS) for this study. Michigan participates in the national Behavioral Risk Factor Surveillance System (BRFSS), which is coordinated by the Centers for Disease Control and Prevention (CDC) and comprises annual, state-level, random-digit–dialed telephone surveys of adults (www.cdc.gov/brfss/). The Office for Survey Research at Michigan State University conducted the 2005 MiBRFS throughout the calendar year among a representative, statewide sample of adults aged 18 years or older, following the CDC BRFSS protocol. The Michigan Department of Community Health Institutional Review Board classified the Michigan BRFSS as nonresearch and determined it to be exempt from review.

The annual MiBRFS questionnaire includes the core CDC BRFSS instrument and state-added questions that focus on Michigan-specific data needs and interests. We developed 12 questions about fast-food consumption on the basis of current literature and 2 sets of pretests and included them as state-added questions in the 2005 MiBRFS. The first question was, "How often do you usually go to a fast-food restaurant?" If response to this initial question was at least once per month, 11 additional questions about fast food were asked (Appendix); if the response was less than once per month, additional questions were skipped because the effect of fast food on the respondent's diet would be minimal. A definition of "fast-food restaurant" was not provided to respondents.

We defined "regular fast-food consumption" as usually going to a fast-food restaurant 2 or more times per week. We used standard BRFSS definitions for obesity (body mass index [BMI] ≥30.0 kg/m^2^, calculated by using self-reported height and weight), fruit and vegetable consumption (<5 times/d vs ≥5 times/d, based on summed responses to 6 fruit and vegetable frequency questions), adequate leisure-time physical activity (≥30 min moderate physical activity ≥5 d/wk or ≥20 min of vigorous physical activity ≥3 d/wk vs not meeting either of these criteria, based on the core CDC BRFSS moderate and vigorous physical activity questions), and general health status (response to the question, "Would you say that in general your health is excellent, very good, good, fair, or poor?")

We used responses to the standard BRFSS demographic questions to define categorical variables for respondents' age, sex, race (3-level variable: white, black, all others), education, household income, and whether there were any children in the household. Urbanicity was based on county-defined metropolitan and micropolitan statistical areas (22). The city of Detroit was classified separately because a state-added question regarding residence in the city of Detroit allowed this distinction.

We restricted our analysis to respondents aged 18 to 64 years to limit the possibility of confounding due to dietary and weight status changes related to poor health among the elderly. We excluded cases with missing fast-food frequency data from all analyses; cases with missing explanatory variables were excluded on an analysis-by-analysis basis.

We used SAS-callable SUDAAN (RTI International, Research Triangle Park, North Carolina) for all statistical analyses to account for the complex survey design and analysis weights that adjusted for the probability of selection and poststratified to the Michigan adult population by age, race, and sex. We calculated prevalence estimates and 95% confidence intervals (CIs) for regular fast-food consumption by the following demographic, socioeconomic, and health-related characteristics: age, sex, race, urbanicity, presence of children in the household, education, annual household income, fruit and vegetable consumption, physical activity, and general health status. We used χ^2^ tests to assess overall differences and generated odds ratios for regular fast-food consumption by using multivariate logistic regression models that adjusted for all demographic, socioeconomic, and health-related variables. We calculated percentage distributions with CIs for behavioral characteristics of fast-food consumption among respondents who reported going to fast-food restaurants at least once per month.

We calculated the prevalence of obesity by frequency of fast-food consumption and used a test for trend to examine statistical significance. In addition, we generated crude and adjusted odds ratios for obesity, using univariate and multivariate logistic regression models.

## Results

The BRFSS Council of American Survey Research Organizations response rate for the 2005 MiBRFS was 51%, which was consistent with the median response rate among all participating states and territories (range, 34.6%-67.4%) (2). The total sample size for this analysis was 4,311.

Among Michigan adults aged 18 to 64 years, we estimated that 12% never went to fast-food restaurants, 9% usually went less than once per month, 29% went at least once per month but less than once per week, 23% went once to less than 2 times per week, 13% went 2 to less than 3 times per week, 7% went 3 to less than 4 times per week, and 8% usually went 4 or more times per week.

The prevalence of regular fast-food consumption was 28% (Table 1). The prevalence decreased consistently with increasing age from 37% of people aged 18 to 24 years to 18% of those aged 55 to 64 years and was higher among men than women (33% vs 23%). The associations of regular fast-food consumption with age and sex remained significant (Wald *F* test, *P* < .001) when tested within a multivariate framework with age, sex, race, urbanicity, children in the household, education, income, fruit and vegetable consumption, physical activity, and general health status as independent variables. Regular fast-food consumption was associated with all 3 health-related variables (fruit and vegetable consumption, physical activity, and general health status). Respondents who reported consuming fruits and vegetables less frequently or engaged in less physical activity were more likely to be regular fast-food consumers. Respondents who reported that their general health status was good were more likely to be regular fast-food consumers compared with those reporting excellent or poor health.

Among respondents who reported going to fast-food restaurants at least once per month, the reason for choosing this type of restaurant was that it was quick and convenient (64%), followed by taste of the food (16%), sociability, and its good value in terms of cost (Table 2). Lunch was the meal most frequently eaten at a fast-food restaurant, followed by dinner. Regular fast-food consumers were more likely to usually order meal packages, super-size options, and take-out, compared with those who went to fast-food restaurants less frequently.

More than 70% of respondents who went to fast-food restaurants at least once per month reported that nutritional information about menu items was available at the fast-food restaurants where they usually ate, 11% reported that it was not available, and 19% didn't know or never noticed whether it was available. Of respondents who were aware that nutritional information was available, most reported having read it, and of those who had read it, approximately one-third said that they used this information when ordering always or most of the time. Putting these responses together, an estimated 16% of the respondents who went to fast-food restaurants used nutritional information when ordering. However, in response to a hypothetical question, most said they would be very or somewhat likely to order "healthier" food items when available.

The prevalence of obesity increased consistently with frequency of fast-food consumption (Table 3). The odds of being obese were approximately 50% higher among those consuming fast-food 2 or more times per week compared with those consuming it less than once per week. After adjusting for potential confounding demographic, socioeconomic, and health-related variables, the odds of being obese were even higher. The adjusted odds of being obese were higher among those consuming fast food 2 to [less than] 3 times per week (60%) and ≥3 times per week (81%) compared with people who consumed them less than once a week.

## Discussion

We estimated that approximately 80% of Michigan adults aged 18 to 64 years went to fast-food restaurants at least once per month and 28% consumed fast food regularly (ie, ≥2/wk). Studies reported in the literature agree that fast food is consumed frequently in the United States and that prevalence of fast food consumption is increasing; however, direct comparisons with our results are difficult because studies have varied by year, population, method of data collection, and question structure. Using data from the US Department of Agriculture's Continuing Survey of Food Intakes by Individuals, Bowman and Vinyard (12) reported that 27% of adults had consumed fast food on day 1 of the survey during 1994 to 1996, representing an increase of 10 percentage points since 1989 to 1991. A 2004 nationwide mail survey estimated that among those who had lost weight (regardless of whether they had been able to keep it off), 56% went to a fast-food restaurant for dinner at least once per week and 22% at least twice per week (16). Another study using data from a community-based sample of women aged 20 to 45 years found that 37% of these women ate fast food 2 or more times per week (15).

We found a strong association between fast-food consumption and obesity prevalence among respondents. Regular consumers of fast food had odds of being obese that were 60% to 80% higher compared with those for people who ate fast food less than once per week. This finding is consistent with the national findings associating fast-food consumption with being overweight (BMI ≥25.0 kg/m^2^) (12); however, it is not consistent with results from a population-based survey of adults in Minnesota, which did not find an association between fast-food consumption and BMI (20). This inconsistency may in part be explained by the difference in definition for fast-food frequency used in each study; the Minnesota analysis used a dichotomous fast-food frequency variable (<1/wk vs ≥1/wk), whereas we used a 4-level variable. In addition, both studies used self-reported body weight and height to calculate BMI, and the known measurement error associated with these self-reported measures may have also contributed to this inconsistency.

Similar to what has been reported in the literature (12,13,20), we found regular fast-food consumption to be consistently associated with age and sex in both the bivariate and multivariate analyses. Younger adults and men showed higher prevalence of regular fast-food consumption. However, after accounting for age and sex, we found no other demographic associations with regular fast-food consumption.

The main reason that Michigan adults go to fast-food restaurants is that they are quick and convenient (64%). Similar results have been reported by Rydell et al (23), using data from a convenience sample of adolescents and adults in the Minneapolis/St. Paul, Minnesota, metropolitan area. Limited time, good taste, eating with friends and family, and cost were the most prevalent reasons among a sample of college students from a large Midwestern university (24). Using a population-based sample of adults in Minnesota, Dave et al (20) found that perceived convenience of fast food and a dislike of cooking were significantly related to frequency of fast-food consumption; however, perceived healthfulness of fast food was not related to its consumption. Our results and those of others suggest that future, more detailed investigations into the reasons people frequent fast-food restaurants and how these reasons may vary among demographic subpopulations could contribute to public health practice by suggesting viable alternatives to fast foods.

The major strength of this study is that it provides unique population-based estimates of fast-food consumption specifically among Michigan adults and confirms that in Michigan, similar to the United States and a few other states for which data are available, food from fast-food restaurants contributes significantly to the population's dietary intake. The limitations of this study include those commonly associated with BRFSS and other random-digit−dialed telephone surveys (2). This may include bias due to noncoverage (exclusion of people who live in households that use cellular telephones only or who do not live in private residences) and nonresponse (BRFSS response rates are approximately 50%). Because the conclusions from this study are based on statistical associations, we are confident that any sources of bias that may be present will not result in a change in these findings. Three other limitations of our study are that 1) the term "fast-food restaurants"  was not defined for the respondents in the survey and, therefore, respondents could have interpreted its meaning in different ways; 2) we did not ask respondents in what form the nutritional information at fast-food restaurants was made available to them; and 3) all data used in this analysis are self-reported. Lastly, our results should be interpreted with caution because of the correlational nature of the associations and because of the limits in identifying all potential confounders in the survey.

Our results indicated that, hypothetically, Michigan adults would choose more healthful menu items at fast-food restaurants if they were available; however, in reality, only 16% reported ever using nutritional information to make menu decisions at fast-food restaurants. Results from a recent study conducted in New Haven, Connecticut, found that restaurant diners consumed 14% fewer calories when item calorie labels were included on the dinner menu and even fewer calories when information on the recommended daily calorie intake for the average adult was also printed on the menu (25). Making nutritional information more readily available and easy to use, including providing easy-to-read calorie labels on menus and menu boards at fast-food restaurants, may be a way to help consumers who are already inclined to use nutritional information when ordering.

## Figures and Tables

**Table 1:** Prevalence and Odds of Regular Fast-Food Consumption^a^ by Demographic, Socioeconomic, and Health-Related Characteristics Among Michigan Adults, Michigan Behavioral Risk Factor Survey, 2005

Characteristic	n^b^	Prevalence		Adjusted Odds Ratio(95% CI)^d^	*P* Value^e^

		% (95% CI)	*P* Value^c^		
Overall	N = 4,311	27.7 (26.1-29.4)	NA	NA	NA
**Demographic**					
**Age, y**					
18-24	279	36.5 (30.7-42.8)	<.001	3.20 (2.17-4.71)	<.001
25-34	648	31.8 (27.8-36.1)		2.06 (1.50-2.83)	
35-44	1,004	29.1 (25.9-32.4)		2.02 (1.51-2.72)	
45-54	1,247	23.8 (21.2-26.6)		1.34 (1.03-1.74)	
55-64	1,133	18.2 (15.8-20.8)		1 [Reference]	
**Sex**					
Male	1,680	33.1 (30.5-35.9)	<.001	1.66 (1.38-2.01)	<.001
Female	2,631	22.5 (20.6-24.5)		1 [Reference]	
**Race**					
White	3,715	26.8 (25.0-28.6)	.06	1 [Reference]	.32
Black	389	34.3 (28.7-40.4)		1.25 (0.86-1.81)	
Other	176	25.9 (18.9-34.3)		0.82 (0.51-1.32)	
**Urbanicity^f^**					
Detroit	196	35.6 (27.6-44.6)	.04	1.69 (0.93-3.08)	.14
Other metropolitan area	3,112	27.5 (25.6-29.5)		1.23 (0.88-1.72)	
Micropolitan area	533	29.5 (25.0-34.4)		1.49 (1.00-2.21)	
Rural	465	22.2 (17.6-27.6)		1 [Reference]	
**Children in household**					
None	2,485	26.2 (24.0-28.5)	.09	1 [Reference]	.73
≥1	1,818	29.2 (26.7-31.7)		0.96 (0.78-1.20)	
**Socioeconomic **					
**Education**					
<High school	253	32.1 (25.4-39.8)	.18	1.16 (0.73-1.84)	.72
High school graduate	1,250	27.5 (24.5-30.8)		1.05 (0.81-1.34)	
Some college	1,323	29.0 (26.0-32.3)		1.14 (0.90-1.43)	
College graduate	1,474	25.4 (22.8-28.1)		1 [Reference]	
**Annual household income, $**					
<20,000	481	25.9 (20.8-31.7)	.69	0.70 (0.48-1.02)	.17
20,000-34,999	700	26.0 (22.1-30.3)		0.77 (0.57-1.03)	
35,000-49,999	653	27.3 (23.1-32.0)		0.82 (0.61-1.10)	
50,000-74,999	835	29.7 (26.0-33.6)		1.00 (0.77-1.29)	
75,000	1,166	28.2 (25.3-31.3)		1 [Reference]	
**Health-related **					
**Fruit and vegetable consumption**					
<5 times/d	3,348	30.0 (28.1-31.9)	<.001	1.54 (1.20-1.99)	<.001
≥5 times/d	963	19.3 (16.2-22.7)		1 [Reference]	
**Physical activity [define in footnote]**					
Inadequate	2,066	31.4 (28.9-33.9)	<.001	1.43 (1.18-1.73)	<.001
Adequate	2,092	24.5 (22.2-26.9)		1 [Reference]	
**General health [define in footnote]**					
Excellent	834	21.8 (18.4-25.5)	<.001	0.57 (0.42-0.75)	<.001
Very good	1,597	27.8 (25.2-30.6)		0.83 (0.67-1.03)	
Good	1,291	33.4 (30.2-36.8)		1 [Reference]	
Fair	436	26.7 (21.6-32.5)		0.69 (0.48-0.99)	
Poor	147	11.1 (6.8-17.7)		0.23 (0.11-0.47)	

Abbreviation: CI, confidence interval.

a Consumed fast food ≥2 times per week based on the question, "How often do you usually go to a fast-food restaurant?"

b Values for each variable may not equal the overall n because of missing data.

c Calculated by χ^2^.

d Adjusted odds ratios from a logistic regression with regular fast-food consumption (≥2 times/wk) as the dependent variable, and all demographic, sociodemographic, and health-related characteristics as the independent variables.

e Calculated by Wald *F* test.

f Urbanicity was based on county-defined metropolitan and micropolitan statistical areas (22).

**Table 2:** Characteristics of Fast-Food Consumption Among Michigan Adults Who Frequent Fast-Food Restaurants,^a^ Michigan Behavioral Risk Factor Survey, 2005 (n = 3,279)

Characteristic	Overall, % (95% CI)	Frequency of Fast-Food Consumption,^b ^% (95% CI)	

		<2 Times/Wk	≥2 Times/Wk
**Main reason for choosing fast food**			
Quick, convenient	63.8 (61.8-65.8)	62.8 (60.4-65.2)	65.6 (61.9-69.0)
Taste of the food	16.4 (14.9-18.1)	16.1 (14.3-18.1)	17.0 (14.3-20.2)
Sociability	8.3 (7.3-9.5)	9.9 (8.6-11.4)	5.4 (3.9-7.3)
Value, cost	6.1 (5.2-7.1)	5.7 (4.7-7.0)	6.7 (5.2-8.6)
**Usual meal eaten**			
Breakfast	6.1 (5.1-7.1)	5.7 (4.6-6.9)	6.8 (5.2-8.9)
Lunch	47.7 (45.7-49.8)	45.9 (43.4-48.3)	51.2 (47.5-54.8)
Dinner	38.4 (36.4-40.5)	40.5 (38.0-42.9)	34.7 (31.3-38.3)
**Usual order**			
Meal package	47.6 (45.5-49.7)	45.4 (42.9-47.9)	51.7 (48.0-55.3)
Super-size option	11.6 (10.2-13.1)	9.4 (7.9-11.1)	15.5 (12.8-18.7)
Take-out	68.1 (66.1-70.0)	66.1 (63.7-68.4)	71.8 (68.4-74.9)
**Where take-out is usually eaten**			
In car or other vehicle	42.6 (40.2-45.1)	43.7 (40.8-46.8)	40.8 (36.6-45.1)
At home	42.8 (40.3-45.3)	45.4 (42.3-48.4)	38.3 (34.1-42.7)
At work or office	13.2 (11.6-15.1)	9.5 (7.8-11.5)	19.6 (16.2-23.4)
**Among those who eat in, usually go with . . .**			
Family	59.0 (54.7-63.1)	66.2 (61.3-70.8)	42.1 (34.8-49.8)
Friends	17.8 (14.5-21.7)	16.5 (12.9-21.0)	19.9 (13.5-28.2)
Self	13.5 (11.0-16.6)	10.5 (7.9-13.8)	21.2 (15.5-28.3)
Co-workers	6.5 (4.6-9.1)	4.1 (2.6-6.5)	12.4 (7.6-19.7)
**Nutritional information**			
Is available	70.8 (68.9-72.6)	69.6 (67.2-71.8)	73.1 (69.8-76.2)
Ever read	68.4 (66.0-70.6)	69.0 (66.1-71.7)	67.2 (63.0-71.2)
Use information when ordering always or most of the time	32.3 (29.6-35.1)	33.6 (30.4-37.1)	29.8 (25.3-34.7)
Very or somewhat likely to order "healthier" items	67.5 (65.5-69.5)	68.4 (66.0-70.7)	65.7 (62.1-69.2)

Abbreviation: CI, confidence interval.

a Reported that they go to fast-food restaurants at least once per month.

b Based on response to the question, "How often do you usually go to a fast-food restaurant?"

**Table 3:** Prevalence and Odds of Obesity^a^ by Frequency of Fast-Food Consumption^b^ Among Michigan Adults, Michigan Behavioral Risk Factor Survey, 2005

**Frequency of Fast-Food Consumption**	Obesity Prevalence,% (95% CI)	Odds Ratio (95% CI)	Adjusted Odds Ratio (95% CI)
< once/wk	24.1 (22.1-26.3)	1 [Reference]	1 [Reference]
1 to <2 times/wk	26.4 (23.2-29.8)	1.13 (0.92-1.38)	1.27 (1.00-1.61)
2 to <3 times/wk	32.2 (27.6-37.1)	1.49 (1.16-1.91)	1.60 (1.21-2.13)
≥3 times/wk	32.9 (28.0-38.1)	1.54 (1.19-1.99)	1.81 (1.35-2.44)

Abbreviation: CI, confidence interval.

a Body mass index (BMI), ≥30.0 kg/m^2^.

b Consumed fast food ≥2 times/wk based on the question, "How often do you usually go to a fast-food restaurant?

c Adjusted odds ratios from a logistic regression with obesity (BMI ≥30.0 kg/m^2^) as the dependent variable, frequency of fast-food consumption as the independent variable, and age, sex, race, urbanicity, children in household, education, household income, general health status, fruit and vegetable consumption, and adequate physical activity as possible confounding variables.

## Appendix: Fast-Food Consumption Module From the 2005 Michigan Behavioral Risk Factor Survey

1. The next questions are about eating out. How often do you usually go to a fast-food restaurant?

1 _ _ Times per day
2 _ _ Times per week
3 _ _ Times per month
444 Less than once per month **Go to Closing Statement**
555 Never **Go to Closing Statement**
777 Don't know
999 Refused

2. When you go to a fast-food restaurant, what is the main reason you choose this type of a restaurant instead of another type?

***Note*: If respondent mentions more than one reason, probe with **"**What is the *main* reason you usually choose a fast-food restaurant?**"
**Read 1-5 only if necessary**
1 Taste of the food, you enjoy going to fast food restaurants
2 Value or cost
3 Convenience, fast service, it's quick
4 Person you are with wants to go
5 Your children like fast-food restaurants
6 Fast-food restaurants are conveniently located, or
0 Some other reason (specify)
7 Don't know
9 Refused

3. When you go to a fast-food restaurant, do you usually eat breakfast, lunch, dinner, or a snack?

1 Breakfast
2 Lunch
3 Dinner
4 Snack
5 All meals, no usual meal
6 Other (specify)
7 Don't know
9 Refused

4. When you go to a fast-food restaurant do you usually order a meal package or individual items?

1 Meal package
2 Individual items
3 Each about half the time
6 Other (specify)
7 Don't know
9 Refused

5. (When you go to a fast-food restaurant . . .) do you usually order any of the "super-size" options that are available?

1 Yes
2 No
6 Other (specify)
7 Don't know
9 Refused

6. (When you go to a fast-food restaurant. . .) do you usually eat in the restaurant or take out?

1 Eat in **Go to Q.7**
2 Take out
3 Each about half the time
6 Other (specify) **Go to Q.7**
7 Don't know **Go to Q.7**
9 Refused **Go to Q.7**

6a. Where do you usually eat your take-out?

1 In the car
2 At home
3 At the office
4 Some other place (specify)
7 Don't know
9 Refused

7. (When you go to a fast-food restaurant . . .) do you usually go with family, friends, coworkers, or by yourself?

***Note:* If respondent gives multiple responses, probe with **"**With whom do you go most often?**"
1 Family
2 Friends
3 Coworkers
4 Self
6 Other (specify)
7 Don't know
9 Refused

8. Sometimes fast-food restaurants have information available about the nutritional contents of their foods. Is this type of nutritional information available at the fast-food restaurants you usually go to?

1 Yes
2 No **Go to Q.11**
6 Never noticed, never looked **Go to Q.11**
7 Don't know **Go to Q.11**
9 Refused **Go to Q.11**

9. Do you ever read this nutritional information at fast-food restaurants?

1 Yes
2 No **Go to Q.11**
7 Don't know **Go to Q.11**
9 Refused **Go to Q.11**

10. How often does this nutritional information help you decide what to order? Would you say . . .

1 Always
2 Most of the time
3 About half the time
4 Sometimes
5 Never
7 Don't know
9 Refused

11. Some fast-food restaurants are including healthier items on their menu. On a usual basis, how likely are you to order healthier food items? Would you say that you are . . .

1 Very likely
2 Somewhat likely
3 Somewhat unlikely
4 Very unlikely, or
5 Neither likely nor unlikely
7 Don't know
9 Refused
